# Improvement in Tear Ferning Patterns of Sheep Tears After Addition of Various Electrolyte Solutions

**DOI:** 10.3389/fmed.2021.721969

**Published:** 2021-11-19

**Authors:** Raied Fagehi, Gamal A. El-Hiti, Bayh M. Alqarni, Mana A. Alanazi, Ali M. Masmali, Turki Almubrad

**Affiliations:** ^1^Department of Optometry, College of Applied Medical Sciences, King Saud University, Riyadh, Saudi Arabia; ^2^Cornea Research Chair, Department of Optometry, College of Applied Medical Sciences, King Saud University, Riyadh, Saudi Arabia

**Keywords:** tear ferning, electrolyte, homogenous mixture, sheep-lamb, grading scale

## Abstract

**Objective:** This study aimed to improve the tear ferning (TF) patterns in the sheep tears after the addition of various electrolyte solutions in different proportions.

**Animal Studied:** Sheep were located at a small farm in the outskirts of Riyadh, Saudi Arabia. The sheep had no ocular disorders or diseases, and none of the female sheep were pregnant.

**Methods:** Tear samples (20 μl) were collected from the right eyes of seven healthy sheep (five female sheep and two male sheep; age 7–36 months with an average of 17.0 ± 10.3 months). A tear sample (1 μl) from each sheep was dried on a microscopic glass slide at 22°C and <40% humidity. The TF patterns were graded based on the five-point grading scale in 0.1 increments. Homogenous mixtures were prepared by mixing tears from each sheep (0.5 μl) with various electrolyte solutions in different proportions (1:1, 1:2, 1:4, 1:6, 1:8, and 1:10). A sample of each mixture (1 μl) was dried on a glass slide, and the TF patterns for each mixture were observed, recorded, graded, and compared with those of the corresponding pure sheep tears. In addition, each sheep tear sample (0.5 μl) was diluted with pure water (0.5 μl) and the TF images were recorded and graded to test the dilution effect.

**Results:** General improvement was noted in TF grades after the addition of electrolyte solutions, ranging from 1.7–1.4 to 1.3–0.3 regardless of the ratio between the electrolyte solutions and sheep tears within the mixture. TF grades of sheep tear samples improved significantly after adding different volumes of calcium chloride solution. Similar improvements in TF grades were observed when magnesium chloride hexahydrate and sodium dihydrogen phosphate solutions were used as the electrolytes. Some improvements in the TF grades occurred with the addition of potassium chloride to sheep tear samples. There was little improvement in TF grades after the addition of sodium chloride solution.

**Conclusion:** Tear ferning grades of sheep tear samples improved when mixed with a number of electrolyte solutions at different volumes, in particular with calcium chloride or magnesium chloride solutions. Some improvements in TF grades were seen with sodium dihydrogen phosphate or potassium chloride solution added as the electrolyte. Clearly, divalent electrolytes lead to a greater improvement in TF grades of sheep tear samples as compared with sodium dihydrogen phosphate or monovalent electrolytes.

## Introduction

Tear film status determines the integrity of the ocular surface (1–3). It protects the eye against microorganism infection, supplies nutrients and oxygen to the cornea, and creates the smooth surface of the eye (4, 5). Excessive evaporation of tears or reduced production of aqueous content leads to instability within the tear film. Such instability results in a common condition known as dry eye (6). Severe dry eye leads to damage to the conjunctiva, ocular epithelia, and cornea (7). In addition, dry eye causes discomfort, visual disturbance, irritation, burning, inflammation, redness, and blurred vision (7). A significant proportion (10–20%) of the global population experiences dry eye symptoms (8, 9). Women and elderly individuals have a high prevalence of dry eye as compared with young and male subjects, respectively (10). Humidity, temperature, smoking, aging, hormone changes, use of digital screens for a long period, contact lens wear, and consumption of alcohol all contribute to the dry eye (11–13). In addition, dry eye is associated with several illnesses such as diabetes, thyroid gland disorders, vitamin A and D deficiencies, high blood cholesterol level, and high body mass index (14–19).

Common tools used to diagnose the dry eye include the Schirmer test (20), phenol red thread (20), tear break-up time (21), osmolarity (22), tear evaporation rate (23), and tear ferning (TF) (24) tests. Alongside other *in vivo* dry eye tests, the TF test has been proven to be simple, repeatable, and useful for assessing the quality of tears (25). The shape of the ferns or the crystallization patterns produced from dried tears is affected by several factors, such as temperature, humidity, and concentration of electrolytes, proteins, and mucins. The interaction between electrolytes (e.g., sodium and calcium chlorides) and macromolecules (e.g., proteins and mucins) plays an important role in the formation of ferns (26). Studies have suggested various mechanisms involved in fern formation (27–29). To grade the quality of tear ferns, TF grading scales are used. The most common scales are the four- and five-point TF grading scales (30, 31). The four-point TF grading scale includes four grades (types I, II, III, and IV) (30). Types I and II represent tears collected from normal healthy eyes, and types III and IV represent dry eye. The five-point TF grading scale includes grades from 0 to 4 in which grades below two represent dry eye (31). Such scales can be converted to 0.1 increments, which make it easy to differentiate between various types of ferns. Both TF grading scales have been used to assess tear ferns in humans and animals (32–38).

The addition of electrolyte solutions to eye drops (e.g., Refresh Plus Tears® and Blink Contact Soothing Eye Drops®) has been reported to improve the TF grades (39). Therefore, the aim of the present study was to assess the effect of the addition of various electrolyte (monovalent, divalent, and hydrogenated) solutions in different proportions on the TF patterns of sheep tears. We expected that the TF patterns of sheep tears would be improved when mixed with electrolyte solutions as a result of the enhancement of the chemical nature of the tears. In addition, the electrolytes used constitute the main components of basic tear solution (40). To the best of our knowledge, this is the first report regarding the improvement in TF grades of sheep tears through the addition of electrolyte solutions.

## Materials and Methods

### Animals

We collected tear samples from seven healthy sheep (two male sheep and five female sheep) ranging in age from 7 to 36 months (17.0 ± 10.3 months). The sheep were located at a small farm in the outskirts of Riyadh City. The sheep had no ocular disorders or diseases, and none of the female sheep were pregnant. Tear samples were collected by the same examiner at the same environment under identical care and nutritional conditions, such as freshwater supply. Microcapillary tubes (50 μl; Merck, Darmstadt, Germany) were used to collect the tear samples. The process of tear collection was performed with care and diligence to ensure that the sheep were not exposed to any level of trauma. No lacrimation instruments or anesthetic was used during the collection of tears. Tear samples were collected from the lower meniscus of the right eye of each sheep. The tears were transferred immediately to Eppendorf tubes, stored in a cooled container, and transported to the laboratory. The samples arrived in the laboratory within 70–80 min from tears collection from sheep and the effect of the addition of the electrolyte solution was investigated immediately.

### Electrolyte Solutions

The salts were obtained from Avonchem Limited (Macclesfield, UK). Solutions of sodium chloride (NaCl; 680 mg), potassium chloride (KCl; 140 mg), calcium chloride (CaCl_2_; 5 mg), magnesium chloride hexahydrate (MgCl.6H_2_O; 12 mg), and sodium hydrogen phosphate (NaH_2_PO_4_; 9.4 mg) were prepared in double-distilled water (100 ml). The mixtures were stirred for 5 min using a Stuart magnetic stirrer (Cole-Parmer, UK) to produce a homogenous solution of each salt. The concentrations of electrolyte solutions were the same as in the basic tear solution (40).

### TF Test

A sample (1 μl) of tears collected from each sheep was dried on a microscopic glass slide at a temperature of 22°C and <40% humidity for 10 min. The TF patterns of the dried tears were observed under an Olympus DP72 microscope (magnification power = 20×) and graded based on a five-point grading scale with 0.1 increments (31). Homogenous mixtures were prepared by mixing tears collected from each sheep (0.5 μl; *N* = 7) and various electrolyte solutions in different proportions (1:1, 1:2, 1:4, 1:6, 1:8, and 1:10 by volume). A sample (1 μl) of each mixture was allowed to dry on a glass slide, and the TF patterns for each mixture were observed, graded, and compared with those of the corresponding sheep tears. For comparison, a tear sample from each sheep (0.5 μl) was diluted with double distilled water (0.5 μl) and the TF patterns were recorded for each mixture. The TF grades of diluted tears were exactly the same as for those before dilution. Three independent examiners graded the TF patterns with the second and third examiners were marked to avoid bias. In many cases, the scores from the examiners were similar. An average TF grade was recorded and rounded to the nearest one decimal place.

## Results

Table 1 lists the age and sex of the seven healthy sheep from which the tear samples were collected. The TF grades of the sheep tear samples ranged from 1.4 to 1.7 based on the five-point grading scale (31). Electrolyte solutions of KCl, NaCl, CaCl_2_, MgCl_2_.6H_2_O, and NaH_2_PO_4_ (0.5–5 μl) were mixed with tears collected from each sheep (0.5 μl). The TF patterns of each homogenous mixture were observed and graded (31). The TF grades of tears diluted with pure water were very similar with those recorded for the pure sheep tear samples. Clearly, pure water has little or no effect on the TF patterns of sheep tears.

**Table 1:** Age and sex of the sheep (*N* = 7) from which tear samples were obtained.

**Tear sample**	**Age (months)**	**Sex**
S1	7	Female
S2	8	Male
S3	8	Male
S4	12	Female
S5	36	Female
S6	24	Female
S7	24	Female

*S1, first sheep tear sample; S2, second sheep tear sample; S3, third sheep tear sample; S4, fourth sheep tear sample; S5, fifth sheep tear sample; S6, sixth sheep tear sample; S7, seventh sheep tear sample*.

**Figure 1:**
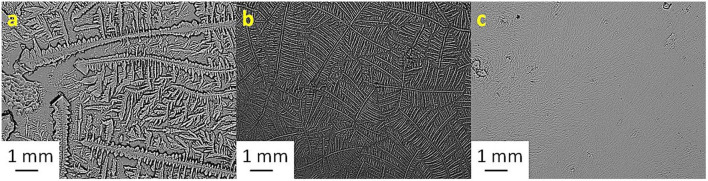
Tear ferning patterns of **(a)** S1, **(b)** S1:KCl (1:10), and **(c)** S1:CaCl_2_ (1:10). S1, first sheep tear sample.

**Figure 2:**
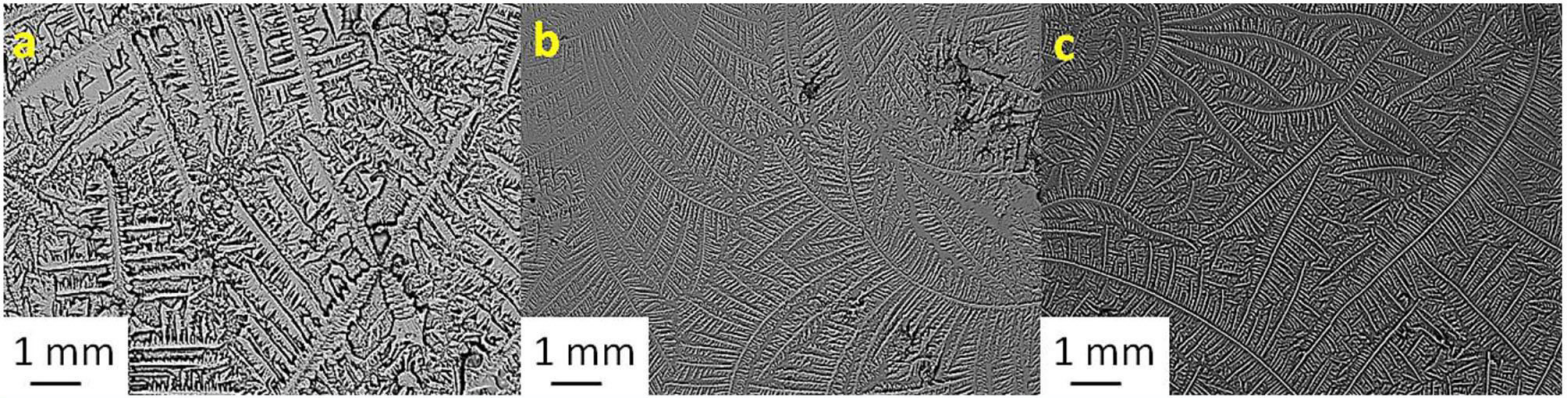
Tear ferning patterns of **(a)** S2, **(b)** S2 and KCl (1:1), and **(c)** S2:MgCl_2_.6H_2_O (1:8). S2, second sheep tear sample.

**Figure 3:**
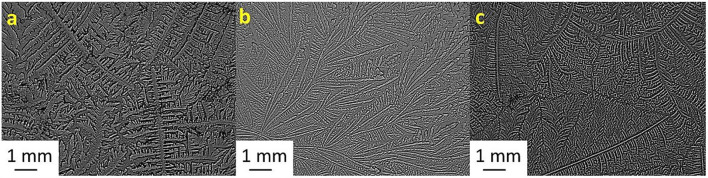
Tear ferning patterns of **(a)** S3, **(b)** S3:NaCl (1:10), and **(c)** S3:MgCl_2_.6H_2_O (1:2). S3, third sheep tear sample.

**Figure 4:**
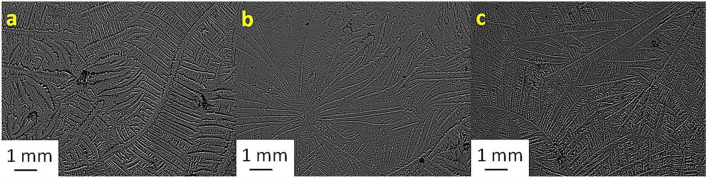
Tear ferning patterns of **(a)** S4, **(b)** S4:NaCl (1:1), and **(c)** S4:KCl (1:8). S4, fourth sheep tear sample.

**Figure 5:**
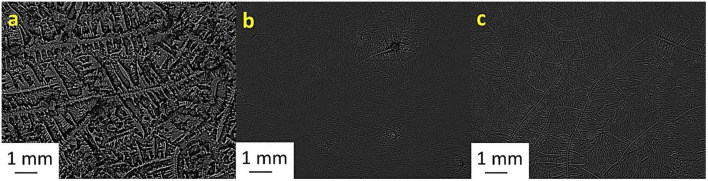
Tear ferning patterns of **(a)** S5, **(b)** S5:MgCl_2_.6H_2_O (1:2), and **(c)** S5:NaH_2_PO_4_ (1:10). S5, fifth sheep tear sample.

**Figure 6:**
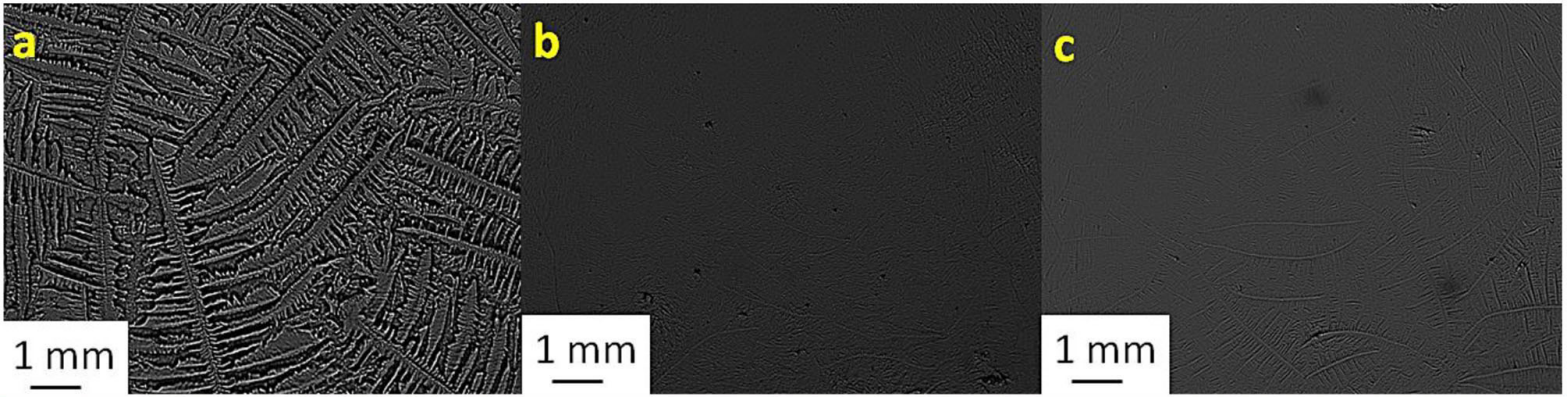
Tear ferning patterns of **(a)** S6, **(b)** S6:CaCl_2_ (1:8), and **(c)** S6:MgCl_2_.6H_2_O (1:10). S6, sixth sheep tear sample.

**Figure 7:**
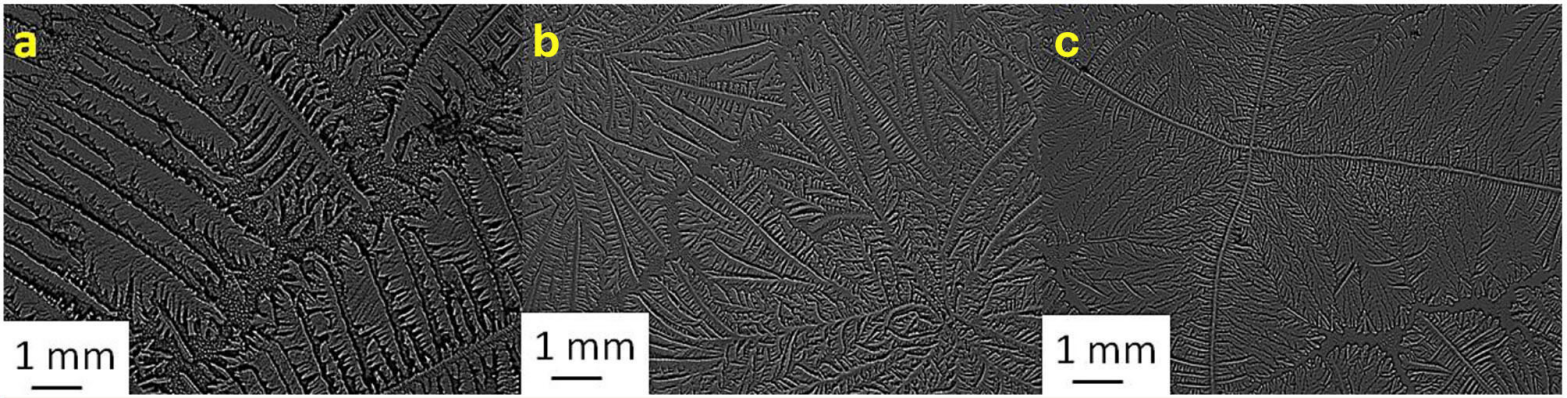
Tear ferning patterns of **(a)** S7, **(b)** S7:KCl (1:2), and **(c)** S7:CaCl_2_ (1:4). S7, seventh sheep tear sample.

Table 2 shows the TF grades for tear samples collected from each sheep and their corresponding mixtures with electrolyte solutions. Figures 1–7 present the examples of TF images of sheep tears and their corresponding mixtures with various electrolyte solutions that led to the most significant improvements.

**Table 2:** Tear ferning grades of sheep tears mixed with various electrolyte solutions.

**Electrolyte**	**Ratio^a^**	**TF grade^b^**						
		**S1**	**S2**	**S3**	**S4**	**S5**	**S6**	**S7**
–	–	1.6	1.6	1.5	1.4	1.7	1.6	1.7
NaCl	1:1	1.5	1.4	1.4	1.3	1.5	1.5	1.6
	1:2	1.4	1.4	1.4	1.6	1.5	1.4	1.5
	1:4	1.5	1.4	1.8	1.6	1.5	1.4	1.4
	1:6	1.5	1.4	1.6	1.5	1.5	1.4	1.4
	1:8	1.5	1.4	1.4	1.3	1.6	1.4	1.4
	1:10	1.5	1.4	1.3	1.3	1.4	1.5	1.4
KCl	1:1	1.5	1.3	1.5	1.4	1.4	1.3	1.3
	1:2	1.5	1.3	1.6	1.2	1.3	1.3	1.2
	1:4	1.5	1.2	1.4	1.3	1.1	1.2	1.2
	1:6	1.5	1.3	1.5	1.3	0.7	1.2	1.3
	1:8	0.9	1.2	1.5	1.2	0.9	1.1	1.3
	1:10	1.2	1.1	1.4	1.2	0.9	1.1	1.3
CaCl_2_	1:1	0.5	1.3	1.3	1.3	1.3	1.3	0.9
	1:2	1.2	1.1	1.4	0.8	1.2	1.2	0.9
	1:4	1.1	1.3	1.3	0.9	0.9	0.9	0.8
	1:6	1.3	1.1	1.4	0.9	0.8	0.9	0.8
	1:8	0.7	1.2	1.3	1.0	0.8	0.7	0.8
	1:10	0.3	1.3	0.6	0.9	0.7	0.8	0.8
MgCl_2_.6H_2_O	1:1	1.3	1.5	1.2	1.1	0.6	1.4	1.6
	1:2	0.8	1.3	1.1	1.2	0.5	1.2	1.4
	1:4	0.8	1.3	1.3	1.1	0.8	1.1	1.4
	1:6	1.1	1.3	1.2	1.2	0.8	1.1	1.4
	1:8	0.9	1.1	1.2	1.1	0.8	1.1	1.1
	1:10	1.2	1.3	1.2	1.1	0.6	0.8	1.4
NaH_2_PO_4_	1:1	1.2	1.5	1.2	1.3	1.4	1.4	1.6
	1:2	1.2	1.5	1.2	1.2	1.4	1.4	1.2
	1:4	1.3	1.5	1.1	1.2	1.3	1.4	1.3
	1:6	1.2	1.5	1.2	1.1	0.8	1.1	1.3
	1:8	1.2	1.2	0.6	0.8	0.8	1.2	1.3
	1:10	1.2	1.3	0.8	0.9	0.7	1.2	1.3

*S1, first sheep tear sample; S2, second sheep tear sample; S3, third sheep tear sample; S4, fourth sheep tear sample; S5, fifth sheep tear sample; S6, sixth sheep tear sample; S7, seventh sheep tear sample*.

a *The ratio of volume (μl) between sheep tear sample (0.5 μl) and electrolyte solution*.

b *Five-point tear ferning (TF) grading scale in 0.1 increments was used for grading. The TF grade was round to the nearest one decimal place for simplicity*.

The results displayed in Table 2 indicated that the TF grades of sheep tear samples generally improved after the addition of electrolyte solutions. The greatest improvement in TF grades was observed with the use of calcium chloride solution, followed by magnesium chloride hexahydrate and sodium dihydrogen phosphate. For example, the TF grades of sheep tear samples were improved from 1.7–1.4 to 1.3–0.3 regardless of the proportion of the electrolyte within the mixture. In fact, the TF grades of sheep tear samples were improved significantly (0.8–0.3) when different proportions of calcium chloride solution were added. A similar improvement in TF grades was observed with the use of magnesium chloride hexahydrate and sodium dihydrogen phosphate solutions as the electrolytes. Some improvements in TF grades were observed when a solution of potassium chloride was added to sheep tear samples. For example, the TF grade of tears collected from one of the sheep was improved from 1.7 to 0.7 with the use of potassium chloride solution (1:6 by volume). However, little improvement in TF grades was observed when sodium chloride solution was added to sheep tear samples. Clearly, solutions of divalent electrolytes (calcium and magnesium chlorides) provide greater improvement in TF grades of sheep tear samples as compared with sodium dihydrogen phosphate (hydrogenated electrolyte) and monovalent electrolytes (sodium and potassium chlorides).

## Discussion

Animal models are useful for the evaluation of medical and surgical interventions for pathogen-caused infections (41). Sheep do not display emotional tears, and tearing is an indication of allergy, irritation of either a physical or chemical nature, chemical spill, corneal ulcer, tear duct blockage, and systemic diseases (42). Because of their large eyes, sheep are a suitable animal model for the *in vivo* testing. In addition, the collection of tear is an easy process (43). Moreover, aqueous secretion of sheep seems to be physiologically similar to that of humans (44).

Normal TF patterns of dried tears are produced as a result of various biochemical processes. TF patterns are mainly dependent on the concentration and type of electrolytes and large molecules present within the tear film (45). The present study proved that the addition of electrolyte solutions to the tears of sheep can significantly improve TF. Such a process is useful *in vitro* test for determining the changes in TF patterns. In addition, it can be used to assess the formulation of new artificial tears. The findings of the present study are in agreement with a pervious study that evaluated the addition of electrolyte solutions to both Refresh Plus Tears® and Blink Contact Soothing Eye Drops® (39).

The previous studies have reported correlations between TF grades (*in vitro*) and scores obtained from other *in vivo* dry eye diagnostic tests as well as questionnaires. These correlations were weak or not statistically different for normal eyes, smokers, diabetes, and subjects with thyroid disorders (13–15, 25). For controlled diabetic subjects, correlations between TF grades and the scores obtained from phenol red thread, tear break-up time, and McMonnies questionnaire were significant, but weak, at −0.349 (*P* = 0.005), −0.374 (*P* = 0.003), and 0.228 (*P* = 0.075), respectively (14). For uncontrolled diabetic subjects, the correlations between TF grades and the scores obtained from phenol red thread and tear break-up time were −0.410 (*P* = 0.001) and −0.539 (*P* < 0.001), respectively (14). An insignificant or weak correlation between TF grades and other dry eye test scores is expected because each test assesses a different parameter or dry eye symptom. Reports have suggested that the TF test is a valid and repeatable *in vitro* technique that can be used alongside other tests to assess tear film (15–19).

For tear samples collected from subjects with normal eyes at different times during the day, the mean difference and 95% limit of agreement for the TF grades was 0.1 ± 0.4 based on the five-point grading scale (25). In the present study, the mean difference in TF grading was < ±0.1 because the grading by two independent examiners was almost identical in most cases. In addition, changes in TF grades were relatively small in some cases.

Tear proteins in animals have been analyzed, and the tears of cow and sheep were found to contain lipocalins. On the other hand, tears of camel and sheep were determined to contain vitelline membrane outer layer protein 1 (VOM1) (1). This protein is absent in human tears (46). VOM1 supports the stability of tear film, and its level might be higher in summer than in winter (46). In addition, tear production might vary based on animal sex and season (47–50). For example, the quantity of tears produced by Merinos lambs and Saanen babies did not differ according to sex, species, and age (*P* > 0.05) (50). On the other hand, adult sheep produce large quantities of tears compared to lambs babies (50). The result of the present study suggests no sex-based differences in the TF patterns among sheep possibly due to the dry climate in Saudi Arabia.

Salts and large molecules in tears help the formation of ferns when dried. The concentration and proportion of mono- and divalent electrolytes play a vital role in shaping the ferns. The electrolytes tend to adjust the osmolarity, reduce tear evaporation, and enhance tear secretion. Indeed, artificial tears improve the tear film stability, the secretion of tears, and the health of the ocular surface and, therefore, can improve the TF patterns (51). As a result, the improvement observed in the TF grade of sheep tears when mixed with electrolyte solutions might be the enhancement of tear film stability and osmolarity. To understand the mechanism by which electrolyte solutions improve the TF grades of tears collected from sheep, a further study is needed. Such research should involve a large number of various electrolytes and sheep. Also, the addition of various types of proteins to sheep tears should be examined. Research in this area could lead to a better management and understanding of dry eye and the production of new artificial tears with better TF grades.

The limitations of the present study are: (1) the small sample size, (2) more female sheep than male sheep, (3) lack of statistical analysis to confirm the significance of the improvements in TF patterns of sheep, and (4) the use of an animal model with no comparison to human tears.

## Conclusion

In conclusion, the TF grades were greatly improved when a calcium chloride or magnesium chloride solution was mixed with tear samples of sheep. Some improvements in TF grades were noted with the addition of a sodium dihydrogen phosphate or potassium chloride solution to tear samples. Clearly, divalent electrolytes lead to a greater improvement in TF grades of sheep tear samples as compared to TF grades obtained by adding a solution of sodium dihydrogen phosphate or monovalent electrolytes.

## Data Availability Statement

The original contributions presented in the study are included in the article, further inquiries can be directed to the corresponding author.

## Ethics Statement

The animal study was reviewed and approved by College of Applied Medical Science Ethical Committee. Written informed consent was obtained from the individual(s) for the publication of any potentially identifiable images or data included in this article.

## Author Contributions

RF: experiment design and data analysis. GE-H: experiment design, writing manuscript, and revision. BA: data collection and writing. MA: writing and revision. AM: tear ferning scale analysis. TA: experiment design, writing, and revision. All authors contributed to the article and approved the submitted version.

## Conflict of Interest

The authors declare that the research was conducted in the absence of any commercial or financial relationships that could be construed as a potential conflict of interest.

## Publisher's Note

All claims expressed in this article are solely those of the authors and do not necessarily represent those of their affiliated organizations, or those of the publisher, the editors and the reviewers. Any product that may be evaluated in this article, or claim that may be made by its manufacturer, is not guaranteed or endorsed by the publisher.
